# Associated effects of lipopolysaccharide, oleic acid, and lung injury ventilator-induced in developing a model of moderate acute respiratory distress syndrome in New Zealand white rabbits

**DOI:** 10.3389/fvets.2025.1477554

**Published:** 2025-03-19

**Authors:** Xingyu Tian, Bin Lu, Yuyan Huang, Wenhao Zhong, Xin Lei, Siyu Liu, Tao Tao, Fengning Yun, Shiyong Huang, Tiqing Tan, Haifeng Liu, Ziyao Zhou, Guangneng Peng, Ya Wang, Kun Zhang, Xiaoli Luo, Zhijun Zhong

**Affiliations:** ^1^Key Laboratory of Animal Disease and Human Health of Sichuan, College of Veterinary Medicine, Sichuan Agricultural University, Chengdu, China; ^2^Department of Pediatric Critical Medicine, Chengdu Women’s and Children’s Central Hospital, School of Medicine, University of Electronic Science and Technology of China, Chengdu, China

**Keywords:** acute respiratory distress syndrome, mechanical ventilation, oleic acid, lipopolysaccharide, animal model

## Abstract

Acute respiratory distress syndrome (ARDS) is a critical pulmonary disorder with manifestations of pulmonary edema, inflammation, and impaired oxygenation. Establishing reliable animal ARDS models has been critical for investigating its mechanisms and for testing pharmacological interventions. The present study sought to induce a moderate ARDS model in New Zealand White rabbits with a model involving a mix of lipopolysaccharide (LPS), oleic acid (OA), and ventilation-induced lung injury (VILI). Four experimental groups were established: negative control (NC, *n* = 4), OA (OM, *n* = 6), LPS + OA (LOM, *n* = 6), and LPS + OA + VILI (LOV, *n* = 6). Throughout the modeling process, vital signs (MAP and HR), respiratory parameters (Cdyn), and hematological indices (WBC and P/F) were continuously monitored, and lung ultrasound was performed. After the experiment, bronchoalveolar lavage fluid (BALF) was collected to measure total protein content, and lung tissue samples were collected to determine the wet-to-dry (W/D) ratio. HE-stained lung tissue sections were prepared and scored according to the ATS guidelines for lung injury scoring. The LOV group showed the most severe lung injury, significantly decreasing MAP and Cdyn. Pathological and ultrasound scores were considerably higher in the LOV group compared to the OM and LOM groups (*p* < 0.05). The lung W/D ratio was significantly higher in the LOM (6.68 ± 0.56) and LOV (7.40 ± 0.56) groups compared to the NC group (5.20 ± 0.16) (*p* < 0.05). At T6, the PaO2/FiO2 ratio in the LOV group was ≤200 mmHg, significantly lower than that in the NC group (*p* < 0.05). Some rabbits in the OM and LOM groups also had PaO2/FiO2 ratios ≤200 mmHg, but the difference compared to the NC group was not statistically significant. In conclusion, this study established a novel moderate ARDS model in New Zealand White rabbits using LPS, OA, and VILI. The model demonstrates severe lung damage, pulmonary edema, and sustained hypoxemia, providing a basis for future research.

## 1 Introduction

Acute respiratory distress syndrome (ARDS) is an acute hypoxemic respiratory failure caused by various pulmonary and extrapulmonary pathological factors other than cardiogenic factors (1). The hallmark characteristics of ARDS include alveolar damage, pulmonary inflammation, and impaired oxygenation (1). ARDS represents one of the most prevalent conditions within the intensive care unit (ICU) (2, 3), with an estimated mortality rate of approximately 40%, affecting millions of individuals annually and resulting in thousands of deaths globally (4).

Animal models serve as a bridge between patients and the laboratory bench, facilitating the direct testing of scientific hypotheses generated in human studies using animal models (5). However, the current widely used murine models have limitations in replicating the development of ARDS in humans (6). The murine lungs have different lobular anatomy from that of humans, with fewer conducting airway branches proximal to the terminal bronchioles (6). Furthermore, most murine models preclude co-interventions and organ support (e.g., vasopressors or mechanical ventilation), continuous sampling across anatomical compartments, or radiological evaluation (7). Given these considerations, the National Heart, Lung, and Blood Institute (NHLBI) advocates using large or medium animal models for ARDS research, rather than murine models (8).

The majority of existing ARDS models have focused on single pathogenic factors, with the result that only some of the pathophysiological characteristics of ARDS can be replicated (9–11). The oleic acid (OA) model is closely associated with the subgroup of ARDS caused by fat embolism, which resembles the acute diffuse lung injury in the initial stage (12). However, the effect of OA is dose-dependent, with high doses often causing severe pulmonary hypertension and right heart failure, which in turn leads to a higher mortality rate in animals (13). The lipopolysaccharide (LPS) model directly acts on vascular endothelial cells to induce an inflammatory response, thereby more closely approximating the conditions observed in ARDS patients (9, 14, 15). However, LPS only causes mild alveolar capillary permeability injury, making it difficult to cause severe hypoxemia and achieve moderate ARDS (16, 17). Mechanical ventilation is a fundamental treatment for ARDS patients, and during the process of mechanical ventilation, ventilation with high strain and stress is necessary to maintain gas exchange for patients (18, 19). It is well-documented that excessive mechanical ventilation can damage healthy lungs and exacerbate existing lung injuries in patients with ARDS (20, 21). Consequently, ventilator-induced lung injury (VILI) is an inevitable consequence of mechanical ventilation (22, 23).

Based on previous studies, we infer that integrating a multifactorial injury model combining LPS-induced endothelial injury, OA-driven alveolar damage, and VILI-mediated mechanical stress will generate synergistic pathophysiological responses, thereby establishing a stable and reliable moderate ARDS model that reproduces diverse clinical manifestations and histological features of human ARDS. By comparing with traditional models (single-hit with OA and dual-hit with OA and LPS), we conducted an in-depth analysis of the similarities and differences in lung injury severity, oxygenation status, and lung ultrasound characteristics, aiming to provide reference for future clinical research.

## 2 Materials and methods

### 2.1 Materials

*Escherichia coli* lipopolysaccharide (serotype O55:B5) (Sigma-Aldrich, St. Louis, MO, United States; Cat# L2880-25MG), Oleic acid (Sangon Biotech, Shanghai, China; Cat# A502071-0250; BC grade, 250 mL), 20% solution of bovine serum albumin (Yeasen Biotechnology, Shanghai, China; Cat#36101ES25; Standard Grade, 25 g), Lowry Protein Assay Kit (Solable, Beijing, China; Cat# PC0030-100T), Mechanical ventilator (Evita4, Dräger, Lübeck, Germany), Ultrasound machine (M9, Mindray, Shenzhen, China).

### 2.2 Experimental animals

All animal experiments were performed according to the guidelines for the care and use of laboratory animals approved by the Institutional Animal Care and Use Committee of Sichuan Agricultural University (Permission number DYY-2022303096). Twenty-two healthy adult New Zealand White rabbits weighing (2.5 ± 0.2) kg were provided by the Experimental Animal Committee Breeding Facility of Sichuan Province [License number: SCXK (Chuan) 2021–037]. The animals were housed in separate wire cages in a room with controlled temperature and humidity, with a 12-h light/dark cycle and were provided free access to tap water and food. Following 7 days of adaptive feeding, the healthy animals were used for subsequent experiments (24).

### 2.3 Animal grouping and model preparation

Based on previous studies, 22 New Zealand White rabbits were selected and numbered sequentially from 1 to 22 according to their body weight in ascending order. The sample size was determined through power analysis, guided by earlier ARDS model research to ensure adequate statistical power (*α* = 0.05 and power = 0.8) for identifying significant group differences, with 4 experimental groups (25). Using Excel 2021, 22 random numbers were generated [formula: =RAND ()], and then the animals were re-ranked according to these random numbers to create a random sequence (Ascending order). Animals were then assigned to groups based on this sequence: the first 4 to the NC group, the next 6 to the OM group, the following 6 to the LOM group, and the final 6 to the LOV group (26). Before the experiment, animals were fasted for 12 h and water-deprived for 6 h. They were transferred to the operating room 1 h before body weight measurement to acclimate to the environment and mitigate stress responses. A 24-gauge intravenous indwelling catheter was placed in the marginal ear vein at this time point (1 h pre-anesthesia). Sequential administration of Telazol (5 mg/kg) through the catheter led to sedation. The quality of the anesthetic action was checked via observation of the lack of withdrawal reflex and stable hemodynamics. Following this, preoperative procedures including hair removal and disinfection were performed under proper restraint. Throughout the modeling process, anesthesia was maintained via continuous infusion of vecuronium bromide (0.06 mg/kg/h) and propofol (6 mg/kg/h) using a syringe pump (BeneFusion SP3D, Mindray, Shenzhen, China). Animals were positioned supine, and a non-cuffed 2.5-mm endotracheal tube was inserted via tracheotomy within 3 s, followed by mechanical ventilation using intermittent positive pressure ventilation (IPPV). Initial ventilator parameters were set as follows: positive end-expiratory pressure (PEEP) of 3–5 cmH_2_O, fraction of inspired oxygen (FIO_2_) at 40%, respiratory rate (RR) of 40 breaths per minute, and tidal volume (Vt) of 10 mL/kg. The arterial partial pressure of carbon dioxide (PaCO_2_) was dynamically monitored using a portable blood gas analyzer (i-STAT 1 300G, Abbott, United States), and respiratory rate was adjusted to maintain PaCO_2_ as close as possible to the range of 35–45 mmHg. Simultaneously, the carotid artery and jugular vein were isolated and cannulated with 24G polyethylene arterial and venous catheters (Smiths Medical, United States). The arterial catheter was connected to a pressure transducer (PT141103, Mindray, Shenzhen, China) via heparinized saline (10 IU/mL)-filled tubing, and the transducer was further connected to a patient monitor (uMEC12, Mindray, Shenzhen, China). Pressure was maintained using a syringe pump to prevent blood reflux and catheter clotting. Both catheters were flushed hourly with heparinized saline (10 IU/mL) to ensure patency. Venous access was retained for drug administration, and the timepoint was recorded as T0. Before drug administration, LPS powder was dissolved in saline solution to yield a stock solution of 1 mg/mL. OA was diluted 10 times with a 20% solution of bovine serum albumin and thoroughly mixed before use. The doses of LPS (750 μg/kg) and of OA (0.06 mL/kg) were established from preliminary experiments, where optimal lung injury and survival rates were achieved. Throughout the experiment, animals received continuous fluid therapy via the marginal ear vein indwelling catheter, consisting of a 2:1 mixture of 0.9% saline and 5% glucose saline, administered at 8–12 mL/kg/h to maintain hydration while avoiding fluid overload (27). Body temperature was maintained between 38 and 39.5°C using ambient operating room temperature control at 25°C, and a heated warming pad (POPOCOLA, Ningbo, China) placed beneath the animal. Emergency medications were prepared for contingency management, including norepinephrine (0.05–0.1 μg/kg/min IV infusion for hypotension) and epinephrine (0.01 mg/kg IV bolus for cardiac arrest resuscitation). Ventilator settings were dynamically adjusted based on real-time physiological parameters (e.g., blood pressure and heart rate).

**Figure 1:**
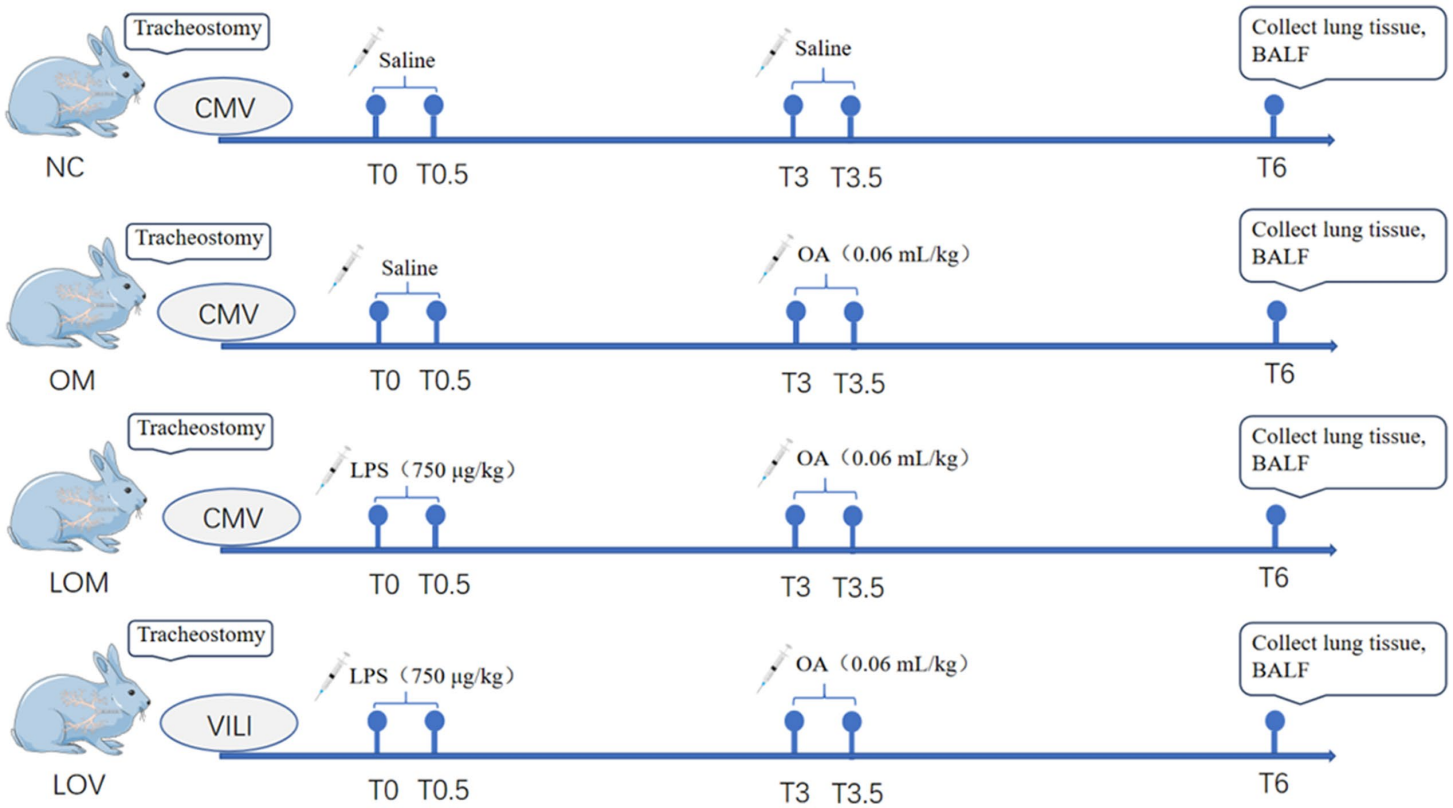
Flowchart of this study. There were four groups in this experiment, and the drug for each animal was completed by infusion over 30 min. NC, OM, and LOM groups maintained conventional ventilation, and LOV group maintained VILI.

The negative control group (NC, *n* = 4) received the same volume of saline as the other groups at T0 and T3, and was maintained on conventional ventilation for 6 h. The OA group (OM, *n* = 6) received saline and OA (0.06 mL/kg) injections via the jugular vein, completed within 30 min at T0 and T3, respectively. Subsequently, conventional ventilation was maintained for 6 h. The LPS + OA group (LOM, *n* = 6) received LPS (750 μg/kg) and OA (0.06 mL/kg) injections via the jugular vein, completed within 30 min at T0 and T3, respectively. Subsequently, conventional ventilation was maintained for 6 h. The LPS + OA + VILI group (LOV, *n* = 6) received LPS (750 μg/kg) and OA (0.06 mL/kg) injections via the jugular vein, completed within 30 min at T0 and T3, respectively. Subsequently, high tidal volume ventilation was maintained for 6 h, and VILI was sustained throughout the process. The ventilator settings employed during the VILI phase were as follows: PEEP of 0 cmH_2_O, FIO_2_ of 40%, RR of 40 breaths per minute, Vt of 15 mL/kg, maintaining PaCO_2_ between 35 and 45 mmHg (Figure 1).

### 2.4 Experimental data collection

The experimental timeline was defined as follows: the pre-surgical phase was designated as T-, the post-surgical stabilization phase as T0, and the entire procedure lasted 6 h. Throughout the experiment, heart rate (HR) and mean arterial pressure (MAP) were continuously monitored and recorded every 10 min (directly acquired from the patient monitor), while body temperature and oxygen saturation were intermittently assessed to maintain normal ranges. Ventilator parameters (PEEP, FIO_2_, RR, and Vt) were dynamically adjusted based on the physiological status. Scheduled measurements included lung dynamic compliance (Cdyn), arterial blood gas (pH, PCO₂, PO₂, SO₂, BE and HCO₃^−^), and lung ultrasound at T0, T3, and T6, with a complete blood count every 2 h (T0, T2, T4, T6). Lung pathological sections were obtained at the experiment’s endpoint. Lung ultrasound examination confirmed the rabbit’s chest had been divided into eight different zones, designated zones 1–8. Each group underwent ultrasound examination in all zones at T0, T3, and T6, and the images were retained for future scoring. All ultrasound assessments were performed on two independent researchers. The scoring criteria are detailed in Table 1 (28).

**Table 1:** Lung ultrasound scoring criteria.

Score	Degree of pulmonary ventilation	Image
0	Normal lung ventilation	Horizontal A-lines, indicative of normal lungs
1	Moderate ventilation loss	Multiple B-lines or the merging of B-lines in localized, delimited areas
2	Severe ventilation loss	Widespread merging B-lines or small areas of consolidation beneath the pleura
3	Complete loss of lung ventilation	Lung consolidation, with or without signs of bronchial inflation

Rabbits were euthanized with an overdose of pentobarbital sodium solution (150 mg/kg, IV) after T6. The bronchoalveolar lavage fluid (BALF) was collected following euthanasia, after which the chest was opened in sterile conditions. The left bronchus was then clamped with hemostatic forceps, and 37°C saline was instilled into the left lung through the tracheal tube in three separate infusions, each with 5 mL, totaling 15 mL. Subsequently, the samples were centrifuged at 2000 rpm for 20 min using a low-temperature centrifuge, after which the supernatant was collected and stored at −80°C for subsequent measurement of protein content in BALF (29). The protein concentration in BALF was then measured using the Lowry Protein Assay Kit.

Collection of pathological tissue samples: Following euthanasia, the upper lobe of the left lung was weighed (wet weight) and then dried in a constant-temperature drying oven at 70°C for 3 days, with the weight recorded (dry weight). The lung wet-to-dry (W/D) ratio was then calculated (16). The remaining tissue from the right lung was fixed in 10% formalin and stained with hematoxylin and eosin (HE) for pathological examination and lung injury pathology scoring. The assessment process conformed to the American Thoracic Society protocols, utilizing precise scoring systems outlined in Table 2. Histological examination was conducted by two independent, group status-blind pathologists with the goal of reducing personal bias (6).

**Table 2:** Lung injury scoring system.

Parameter	Score per field		
	0	1	2
A. Neutrophils in the alveolar space	None	1–5	>5
B. Neutrophils in the interstitial space	None	1–5	>5
C. Hyaline membranes	None	1	>1
D. Proteinaceous debris filling the airspaces	None	1	>1
E. Alveolar septal thickening	<2x	2x-4x	>4x

Score = [(20*A) + (14*B) + (7*C) + (7*D) + (2*E)]/100. The lower lobe of the left lung was taken for HE staining, and five sections were intercepted according to the direction of gravity. Each slice was divided into four regions: upper left, upper right, lower left and lower right. Each region randomly selected five horizons to pathological scoring (400x).

### 2.5 Moderate ARDS success criteria

The success criteria for moderate ARDS were based on the recently published global definition of ARDS in 2023 and subsequently adjusted as required (30).

(1) 100 mmHg<PaO_2_/FiO_2_ ratio ≤ 200 mmHg or 148 < SpO_2_/FiO_2_ ratio ≤ 235 mmHg, with SpO_2_ ≤ 97%.
(2) A reduction in Cdyn by a minimum of 30% compared to the baseline.
(3) The presence of bilateral B-lines and/or consolidation on ultrasound, which cannot be wholly attributed to pleural effusion, atelectasis, or nodules/masses.
(4) Incomplete pulmonary edema or primarily attributable to cardiogenic pulmonary edema/fluid overload, with low oxygenation/blood gas exchange abnormalities not primarily attributable to atelectasis.
(5) The onset or worsening of acute respiratory failure with hypoxemia within 1 week of predicting risk factors or the appearance of new or worsening respiratory symptoms.

### 2.6 Statistical analysis

The data were subjected to analysis using the survival data for each group. Statistical analysis was performed using the SPSS 25.0 software. The results are expressed as means ± SD. Normality was evaluated using the Shapiro–Wilk test. Normal distribution of data was compared by one-way ANOVA with Bonferroni’s *post-hoc* test or Tamhane’s T2 post-hoc test where appropriate. Data within groups were analyzed by One-Way Repeated Measures ANOVA. Kruskal-Wallis analysis was used to compare non-normal data. In this study, a *P*-value <0.05 was considered statistically significant and a *P*-value <0.01 was considered highly statistically significant (31).

## 3 Results

### 3.1 Changes in results of mean arterial pressure, heart rate, and white blood cell count

Table 3 shows the mean values for changes in MAP and HR. In the NC group, the MAP and HR remained stable throughout the experiment, and no signs of respiratory difficulty were observed. Conversely, experimental groups OM, LOM, and LOV had a significant decrease in MAP after T3. No statistically significant differences in MAP changes were observed within the OM group over 6 h. However, in the LOM group, there was a statistically significant decrease in MAP at T6 (*p* < 0.05), while in the LOV group, the decrease in MAP was extremely significant at T6 (*p* < 0.01). The total HR of the rabbits in the OM, LOM, and LOV groups was observed to be lower than that of the NC group. At 1.5 h, the HR of the LOV group was found to be significantly lower than that of the NC group (*p* < 0.05), while no statistical differences were observed between the other two groups and the NC group. At T6, the HR of both the OM and LOV groups was significantly lower than that of the NC group (*p* < 0.05), while there was no statistically significant difference in HR between the LOM group and the NC group. Table 4 shows the mean values for changes in white blood cell (WBC) count in all groups. In all groups, the WBC count exhibited a declining trend at T0, followed by a gradual increase after T2. Compared to the NC group, the OM, LOM, and LOV groups demonstrated an extremely significant difference in WBC count at T2, T4, and T6 (*p* < 0.01).

**Table 3:** Changes results of MAP (mmHg) and HR (bpm).

	Groups			Time (h)		
Items		T0	T1.5	T3	T4.5	T6
MAP	NC	93.50 ± 11.56	93.50 ± 4.93	93.25 ± 4.57	90.25 ± 8.42	92.25 ± 5.25
	OM	89.60 ± 13.65	94.40 ± 4.39	83.40 ± 14.43	84.80 ± 14.60	77.80 ± 12.15
	LOM	94.00 ± 2.00	83.80 ± 6.98	90.00 ± 10.79	81.00 ± 10.37	76.80 ± 11.84*
	LOV	101.00 ± 5.60	97.70 ± 12.34	96.00 ± 5.23	85.50 ± 6.61	69.50 ± 10.21**
HR	NC	263.70 ± 11.62	280.50 ± 21.76	257.25 ± 10.01	267.50 ± 14.39	267.75 ± 10.53
	OM	238.00 ± 18.49	236.80 ± 39.85	234.40 ± 41.10	226.20 ± 15.27**	207.60 ± 28.02**
	LOM	247.00 ± 27.17	255.80 ± 31.41	241.20 ± 26.80	235.80 ± 29.17*	244.80 ± 40.41
	LOV	255.00 ± 36.84	226.25 ± 22.19*	216.25 ± 15.65	217.25 ± 17.54**	222.75 ± 22.5*

Compared to the NC group, *P < 0.05, **P < 0.01.

**Table 4:** Changes results of WBC count (10^9/L).

Groups			Time (h)		
	T-	T0	T2	T4	T6
NC	10.68 ± 2.43	2.98 ± 0.91	2.45 ± 1.31	3.80 ± 1.12	5.20 ± 0.22
OM	9.72 ± 2.00	2.52 ± 0.29	0.98 ± 0.29**	0.98 ± 0.26**	1.94 ± 1.01**
LOM	8.76 ± 1.88	2.58 ± 1.32	0.86 ± 0.31**	0.68 ± 0.20**	1.28 ± 0.48**
LOV	10.25 ± 1.20	2.08 ± 0.51	0.83 ± 0.52**	1.00 ± 0.32**	1.83 ± 1.06**

Compared to NC group, *P < 0.05, **P < 0.01.

### 3.2 Pathological changes in lung tissues

**Figure 2:**
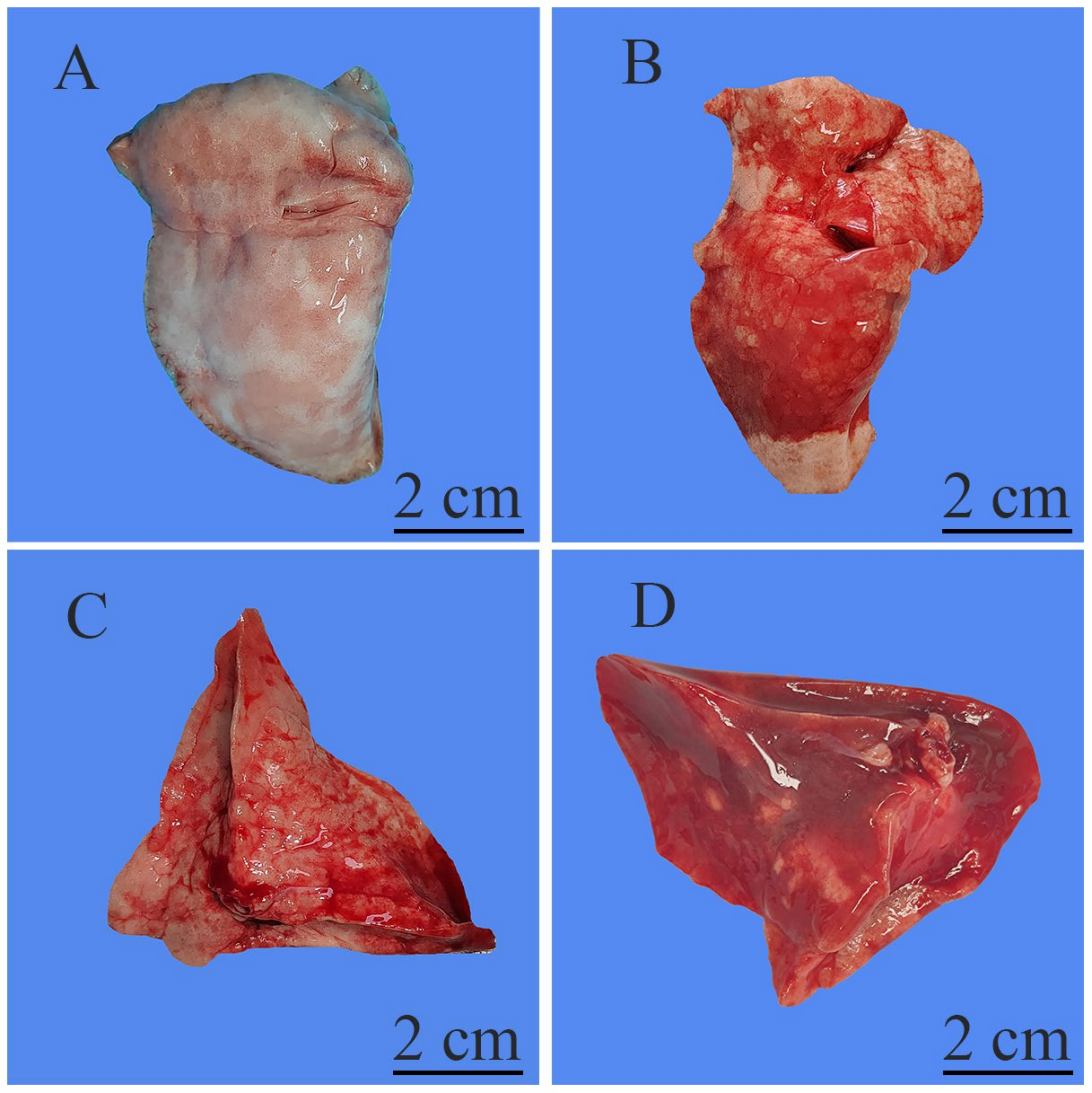
Representative macroscopic images of the lung tissue. **(A)** NC group, normal morphology and size of lung tissues with no obvious lesions. **(B)** OM group, lung tissues showing significant congestion and edema, with patchy hemorrhage on the surface. **(C)** LOM group, lung tissues showing significant congestion and edema, with patchy hemorrhage on the surface. **(D)** LOV group, lung tissues showing severe congestion and edema, with large areas of diffuse hemorrhage on the lung surface, along with a small amount of focal consolidation and necrosis.

**Figure 3:**
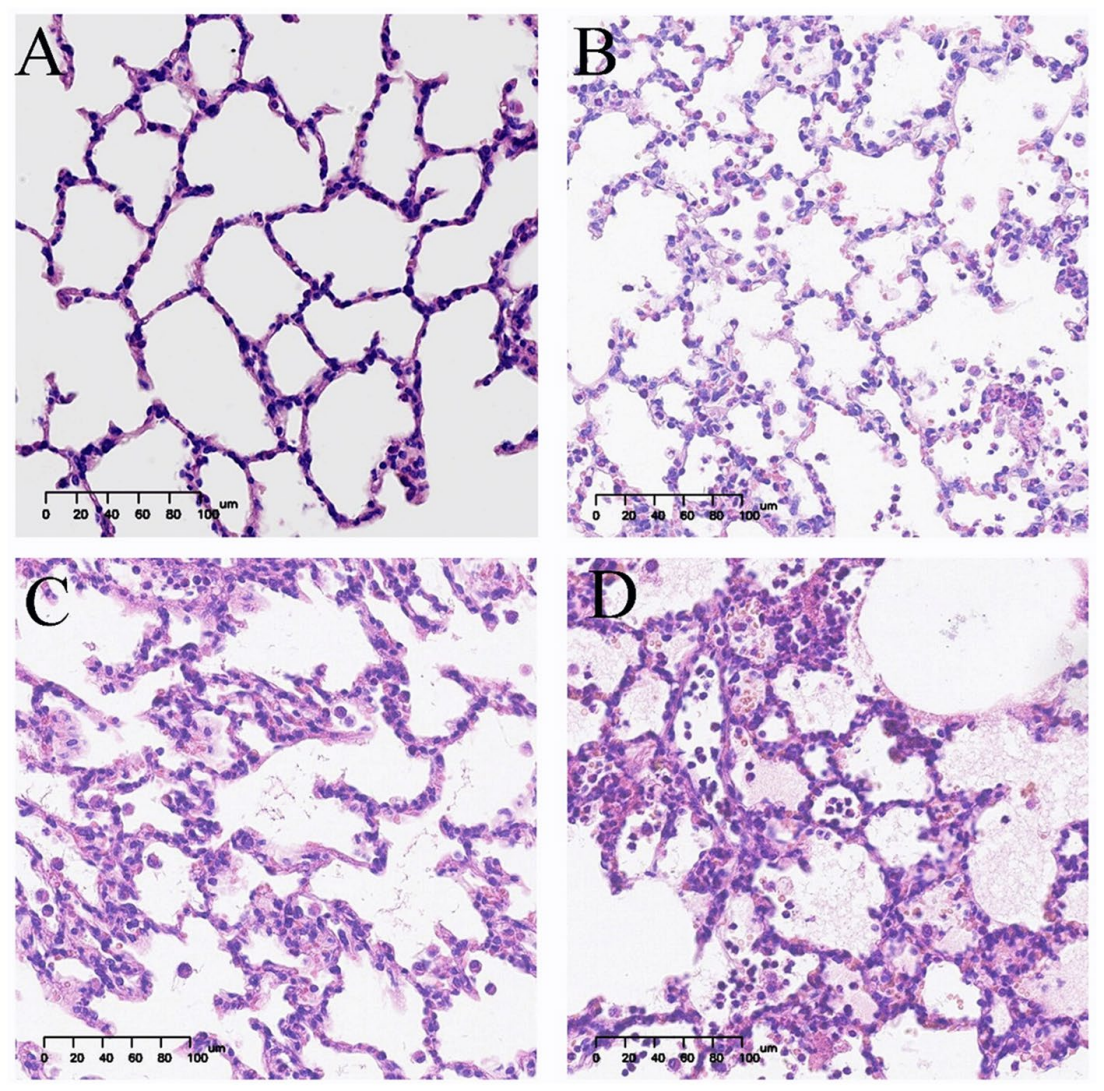
Representative light microscopic images of pathological lung tissue sections (HE staining). Image magnification 200x. **(A)** NC group, intact alveolar structure, clear alveolar space without secretions, and uniform alveolar septa without apparent thickening. **(B)** OM group, thickening of alveolar septa, a small amount of inflammatory cell infiltration and massive exudation of proteinaceous edema fluid. **(C)** LOM group, thickening of the alveolar septa, a small amount of inflammatory cell infiltration and massive exudation of proteinaceous edema fluid. **(D)** LOV group, thickening of alveolar septa, extensive infiltration of red blood cells and inflammatory cells, exudation of proteinaceous edema fluid, and numerous pulmonary bulla.

**Figure 4:**
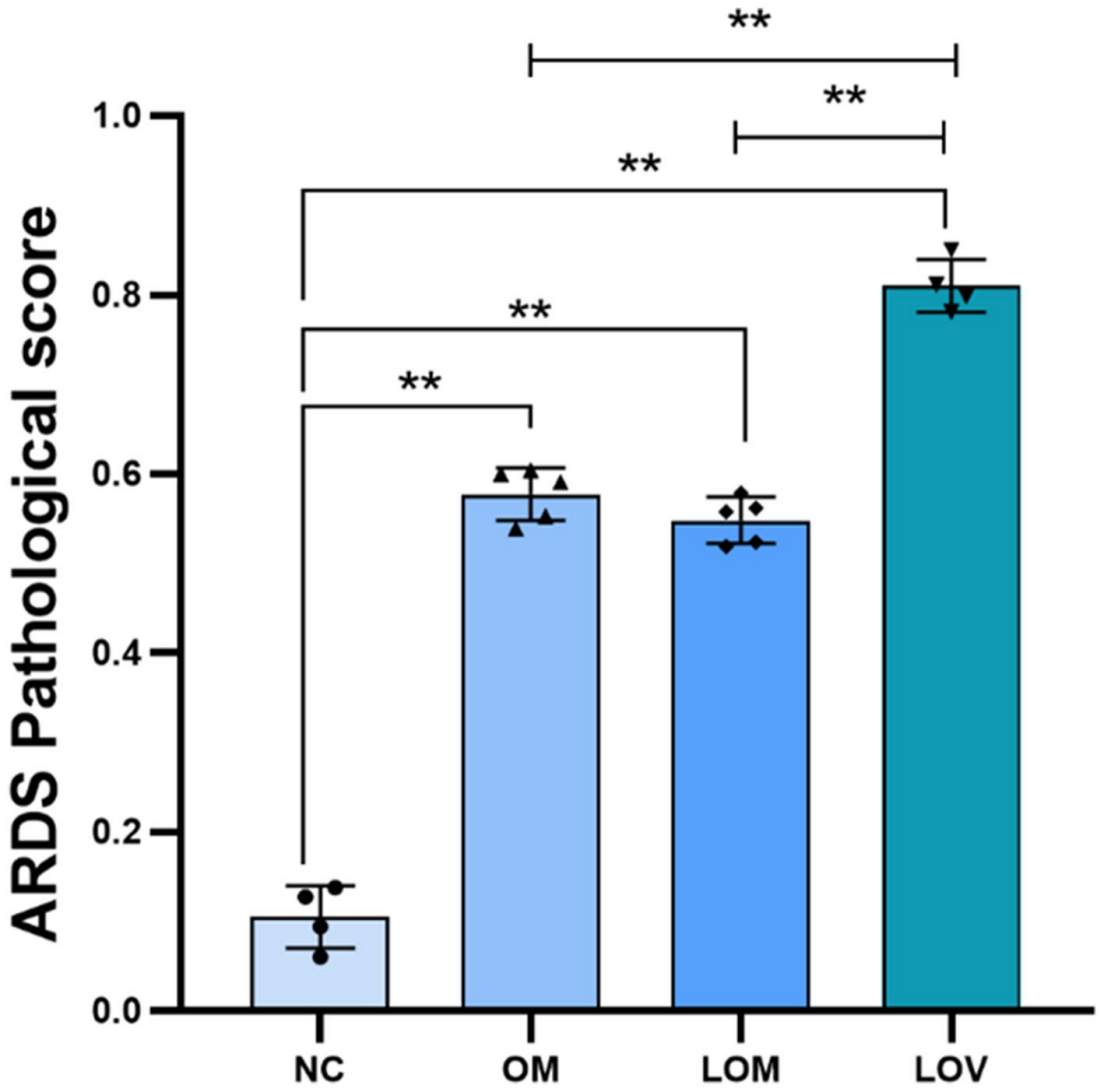
ARDS pathological score of lung tissues. *p < 0.05, **p < 0.01.

Figures 2, 3 show the pathological alterations observed in the lung tissues. In the NC group, the lung tissue exhibited a clear and intact structure, with no evidence of inflammatory cell infiltration, hemorrhage or thickening of the alveolar septa. In the OM group, lung tissue had pronounced congestion and edema with a bright red color. The microscopic study indicated thickened partial alveolar septa, accompanied with accumulation of neutrophils, edema with raised protein levels in the alveolar spaces, red blood cells’ accumulation, and sporadic occurrence of neutrophils. In the LOM group, the lung tissues also exhibited macroscopically evident congestion and edema, appearing bright red. The partial alveolar septa were observed to be thickened, with neutrophil infiltration evident within the alveolar septa and alveolar spaces at the microscopic level. The presence of macrophages and lymphocytes within the alveolar spaces was minimal, accompanied by a notable accumulation of edema fluid and proteinaceous debris, and the localized appearance of hyaline membranes. Within the LOV group, significant hemorrhage and edema of pulmonary tissues were present, with a very intense red color. There was significant thickening of the alveolar septa, with heavy infiltration of neutrophils into the alveoli, red blood cell exudation, and pulmonary bullae formation. Proteinaceous debris and deposition of hyaline membranes were also very significantly more prominent compared with other treatment groups. Furthermore, the experimental groups showed significantly higher scores (*p* < 0.01) than the NC group. The LOV group had the highest score, which was markedly distinct from both the OM and LOM groups (*p* < 0.01). No significant difference was observed between the OM and LOM groups, as illustrated in Figure 4.

### 3.3 Lung tissue wet-to-dry ratio and bronchoalveolar lavage fluid protein content

**Figure 5:**
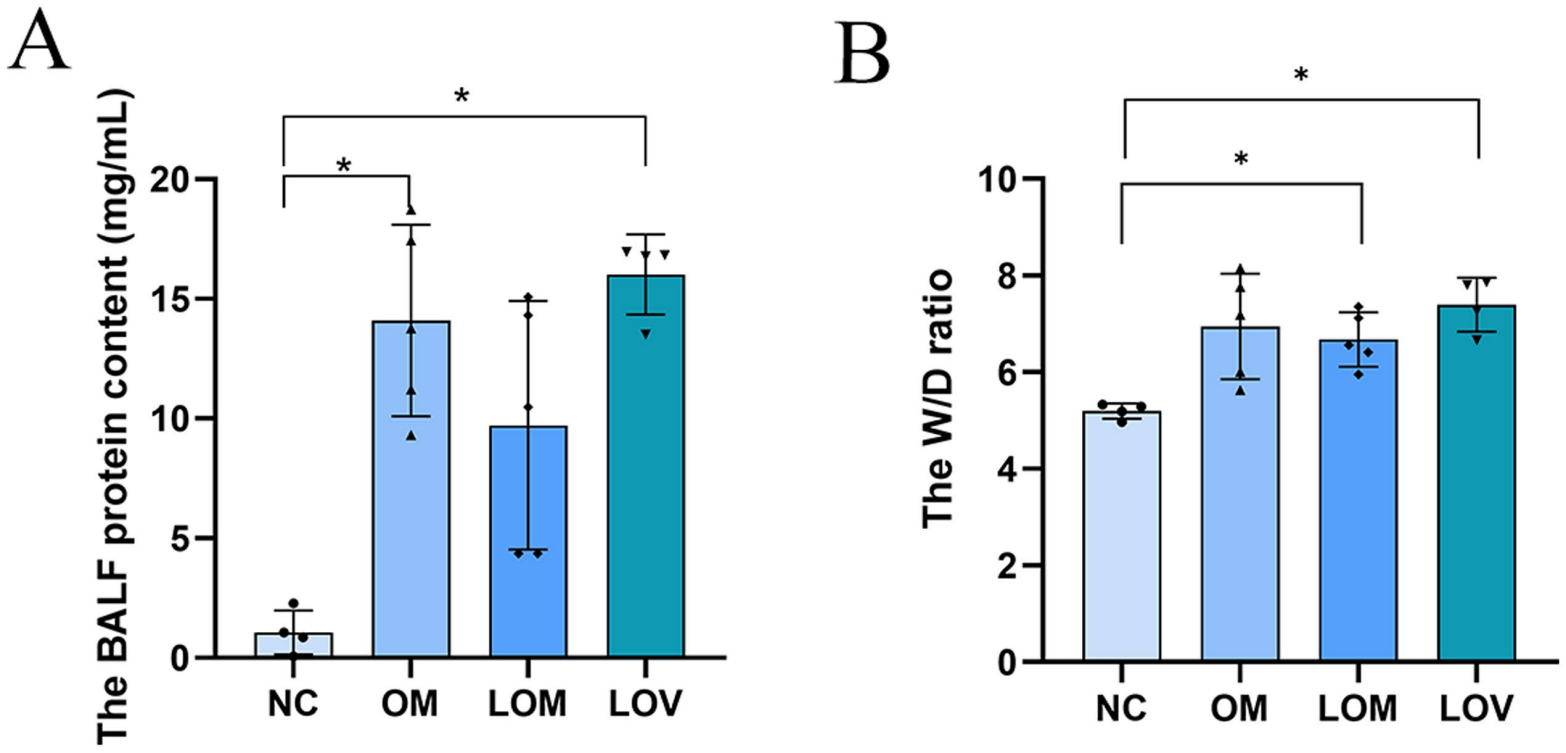
Lung tissue W/D ratio **(A)**. BALF protein content **(B)**. Compared to NC group, *p < 0.05, **p < 0.01.

The concentrations of BALF of the subjects in the OM and LOV groups were significantly higher compared with those of the NC group (*p* < 0.05). The LOM group, on the other hand, had a tendency toward higher BALF levels, with the difference from the NC group failing statistical significance. A comparative study between different groups indicated that the W/D ratio for the LOM group (6.68 ± 0.56) and LOV group (7.40 ± 0.56) was greater compared with the NC group (5.20 ± 0.16) (*p* < 0.05). The OM group also had a rise in the W/D ratio, though this rise failed to achieve statistical significance compared with the NC group (Figure 5).

### 3.4 Lung dynamic compliance

At T3, the LOV group had a significant reduction in lung dynamic compliance (Cdyn) compared with the NC group (*p* < 0.05). Conversely, the OM and LOM groups had declining trends, and fluctuations in the NC group with regard to this parameter did not achieve statistical significance. At T6, a significant reduction was evident in all three experimental groups compared to the NC group (*p* < 0.01). Intra-group comparisons revealed that the Cdyn of the NC group remained stable, while the other three groups exhibited a decreasing trend. The Cdyn of the OM, LOM and LOV groups at T6 was significantly lower than that at T0 (*p* < 0.01), whereas only the Cdyn of the LOV group at T3 was significantly lower than that at T0 (*p* < 0.05), as illustrated in Table 5.

**Table 5:** Changes results of Cdyn (mL/cmH_2_O).

Groups		Time (h)	
	T0	T3	T6
NC	2.63 ± 0.36	2.85 ± 0.17	2.78 ± 0.36
OM	2.89 ± 0.44	2.50 ± 0.25	2.06 ± 0.22**^##^
LOM	2.90 ± 0.45	2.40 ± 0.45	2.02 ± 0.31**^##^
LOV	3.21 ± 0.50	2.34 ± 0.33*^#^	1.71 ± 0.25**^##^

Compared to NC group, *p < 0.05, **p < 0.01. Compare T0 with T3 and T6 separately for each of the four groups, ^**#**^*p* < 0.05, ^**##**^*p* < 0.01.

### 3.5 Base excess and PaO_2_/FiO_2_

**Figure 6:**
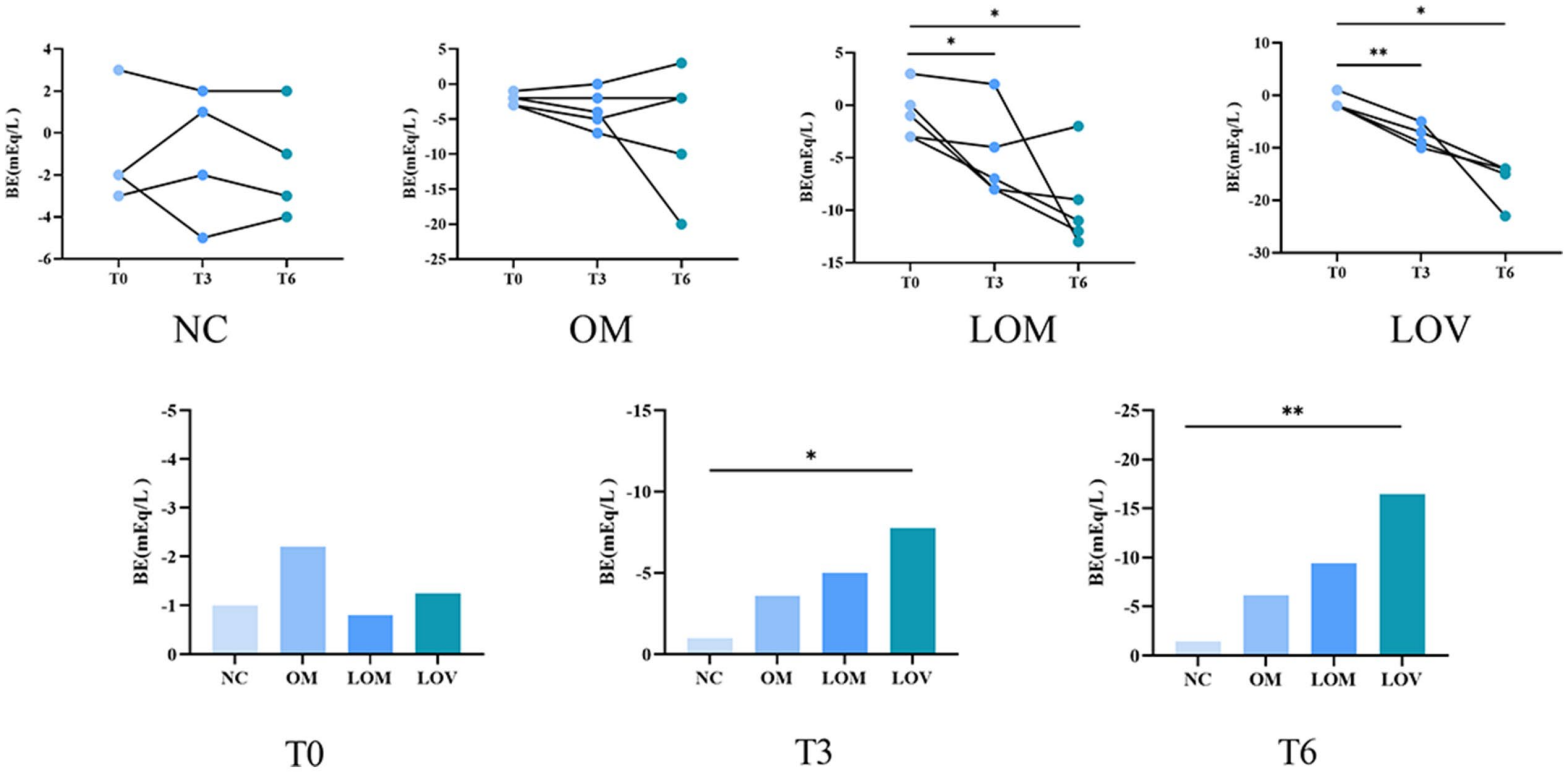
Trends of changes in BE during experiment. *p < 0.05, **p < 0.01.

Intra-group comparisons revealed stable base excess (BE) values throughout the experiment in the NC group, with no significant changes observed. In both the LOM and LOV groups, BE values at T3 and T6 were significantly lower than at baseline (T0) (*p* < 0.05). A subset of animals in the OM group exhibited decreased BE values at T6, although these changes did not reach statistical significance. Inter-group comparisons demonstrated that at T3, the LOV group showed significantly lower BE values than the NC group (*p* < 0.05). At the same time, no significant differences were observed between the OM/LOM groups and the NC group. At T6, BE values in the LOV group were significantly reduced compared to the NC group (*p* < 0.01). In contrast, the OM and LOM groups remained statistically indistinguishable from the NC group, as illustrated in Figure 6.

**Figure 7:**
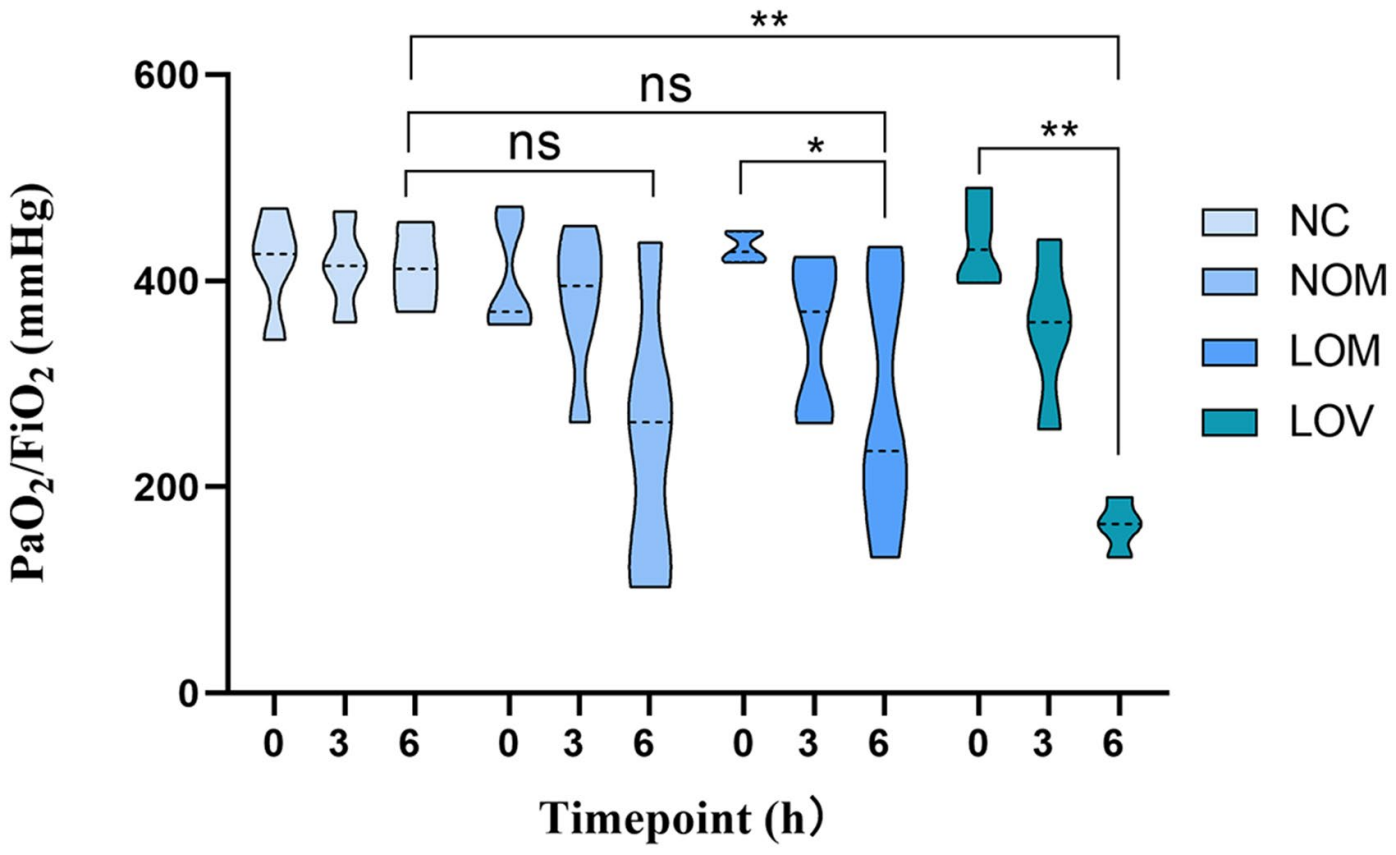
Trend of changes in PaO_2_/FiO_2_ ratio during experiment. *p < 0.05, **p < 0.01.

A comparison of the four groups at T6 revealed that the LOV group had 4 rabbits with P/*F* ≤ 200 mmHg, significantly fewer than that observed in the NC group (*p* < 0.01). The OM and LOM groups had 2 and 1 rabbits with P/F ≤ 200 mmHg, respectively, with no statistically significant difference compared to the NC group. Intra-group comparisons revealed that the PaO_2_/FiO_2_ ratio remained stable throughout the process in the NC group, whereas the other three groups exhibited a declining trend. A comparison of PaO_2_/FiO_2_ at T6 to T0 within each model group revealed that the LOM group showed significantly lower PaO_2_/FiO_2_ at T6 (*p* < 0.05), with a more pronounced decline observed in the LOV (*p* < 0.01). In contrast, the OM group demonstrated no statistically significant difference, as illustrated in Figure 7.

### 3.6 Lung ultrasound scoring and modeling outcomes

**Figure 8:**
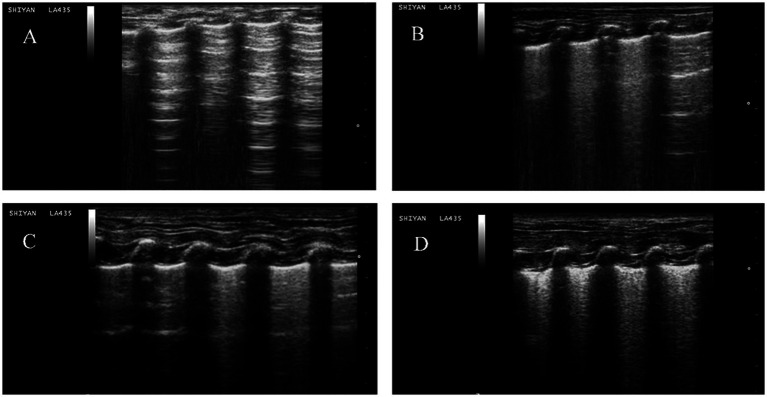
Representative lung ultrasound images for each group. **(A)** NC group, A-lines accompanying pleural sliding. **(B)** OM group, multiple spaced B-lines, with local A-lines. **(C)** LOM group, multiple spaced B-lines, with local partially coalescent B-lines. **(D)** LOV group, multiple coalescent B lines, substantial echogenic areas with hyperechoic dot-like patterns, and static or dynamic signs of bronchial inflation.

**Figure 9:**
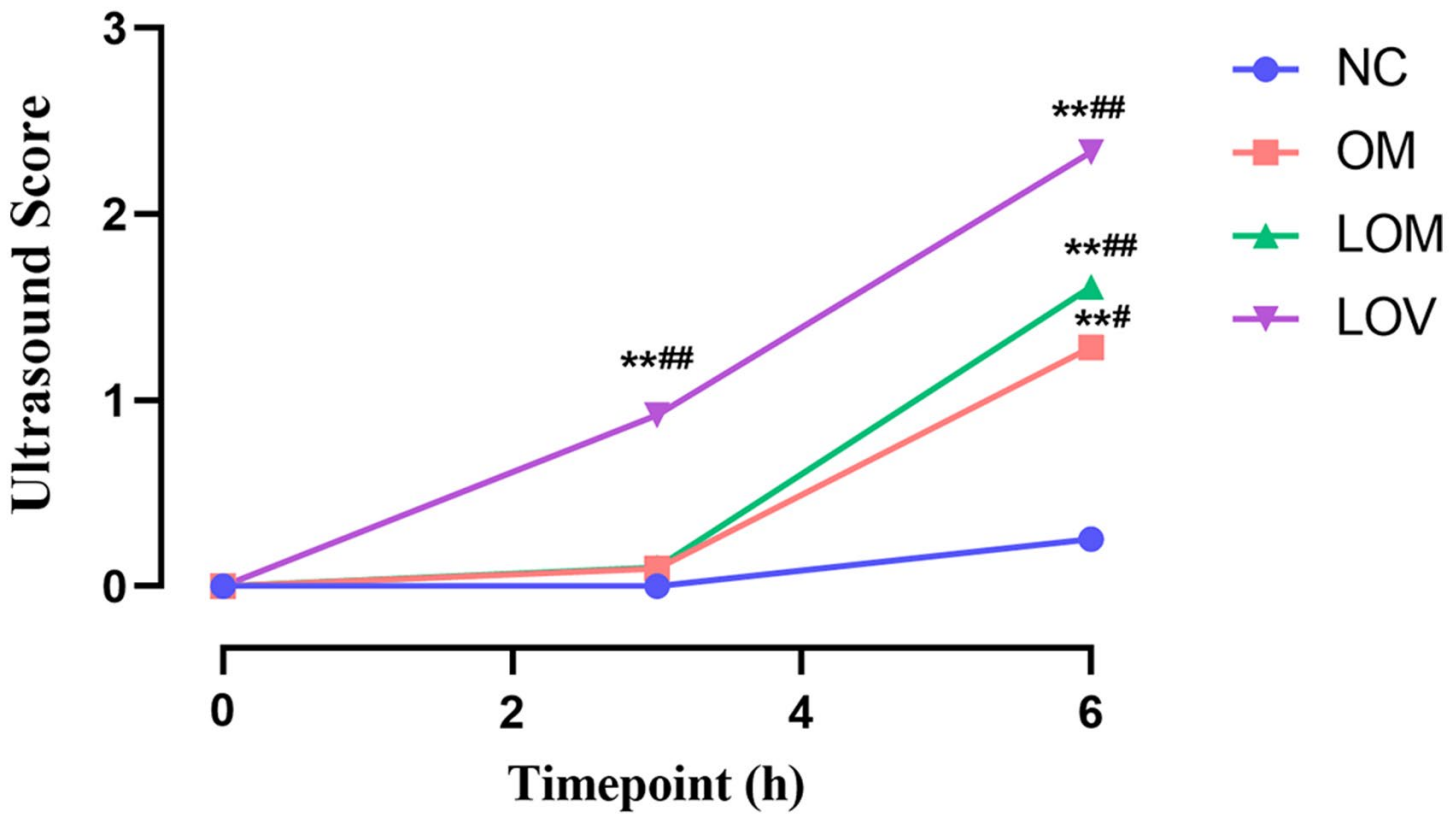
Temporal trend of lung ultrasound scores. Compared to NC group, *p < 0.05, **p < 0.01. Compare T0 with T3 and T6 separately for each of the four groups, ^**#**^*p* < 0.05, ^**##**^*p* < 0.01.

**Figure 10:**
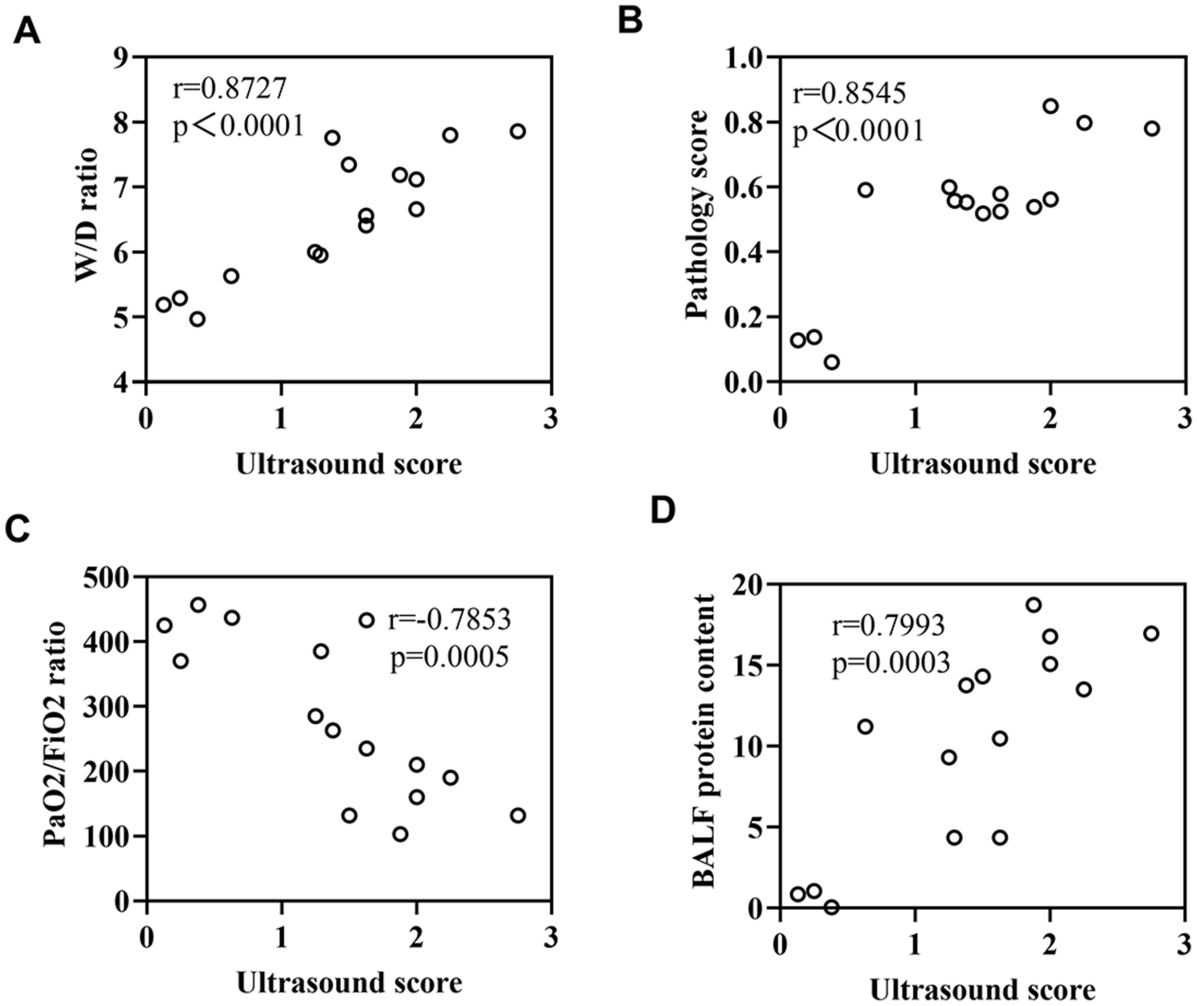
**(A)** Correlation between lung ultrasound score and W/D ratio. **(B)** Correlation between lung ultrasound score and pathological score. **(C)** Correlation between lung ultrasound score and PaO_2_/FiO_2_ ratio. **(D)** Correlation between lung ultrasound score and BALF protein content.

A comparison of the four groups at T3 revealed that the lung ultrasound score of the LOV group was significantly higher than that of the NC group (*p* < 0.01). In contrast, the OM and LOM groups showed no statistically significant difference compared to the NC group. At T6, the lung ultrasound scores in the OM, LOM and LOV groups were significantly higher than that of the NC group. Intra-group comparisons showed that the lung injury worsened over time in the OM, LOM and LOV groups. A comparison of T6 to T0 showed that the OM group had a significantly elevated lung injury (*p* < 0.05), while the LOM group demonstrated a markedly pronounced increase in lung injury (*p* < 0.01). The LOV group exhibited a markedly elevated degree of lung injury at both T3 and T6 compared to T0 (*p* < 0.01), as illustrated in Figures 8, 9. Furthermore, there was a strong correlation between lung ultrasound score and the W/D ratio, pathological score, PaO_2_/FiO_2_ ratio, and BALF protein content, as shown in Figure 10.

**Figure 11:**
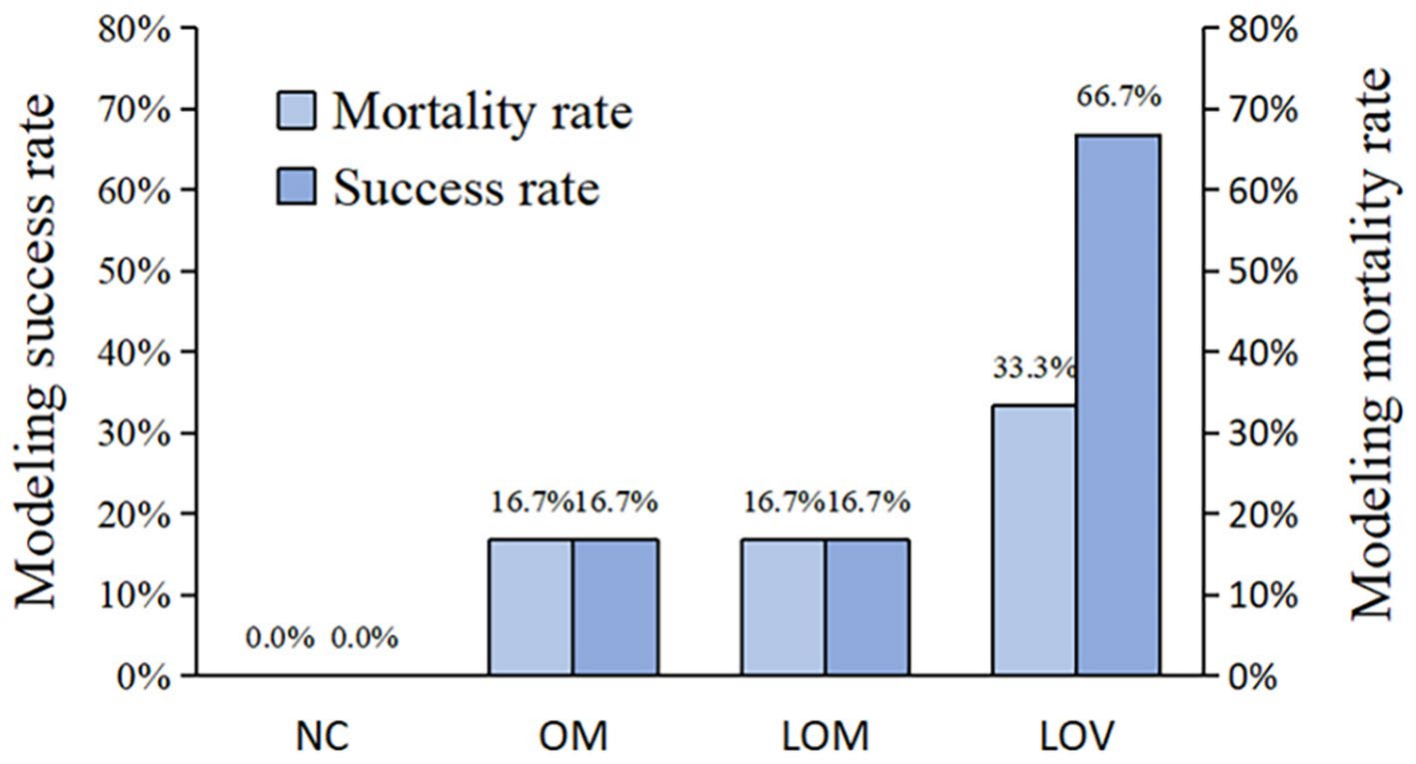
Modeling results of mortality rate and success rate.

According to standard requirements for measuring the severity of ARDS, the rates of modeling success were 16.7% for the OM group, 16.7%% for the LOM group, and 67.6% for the LOV group, and hence, the use of VILI significantly improved the reliability of inducing ARDS of moderate severity. As shown in Figure 11, the success rate in the LOV group was higher than that in the OM and LOM groups.

## 4 Discussion

ARDS is a common disease in the ICU with a high incidence and mortality rate, which has received increasing attention, particularly with the outbreak of COVID-19 (32, 33). Animal models made a significant contribution to our understanding of the pathogenesis and pathophysiological characteristics of ARDS. However, it is essential to note that no animal model can fully replicate all the features of human ARDS (5). In this study, we utilized New Zealand White rabbits to successfully establish moderate ARDS models by employing dual-hit with OA and LPS combined with VILI.

The pathological features of human ARDS include pulmonary neutrophil infiltration, interstitial and alveolar edema, septal thickening, and hyaline membranes (6). In our study, the LOV group exhibited the typical pathological features of ARDS, with a significantly higher pathological score than that observed in the OM and LOM groups (*p* < 0.01). Compared to the OM and LOM groups, the LOV group exhibited a more severe degree of vascular and airway injury (hemorrhage), inflammation (a considerable number of neutrophils and macrophages infiltration), and alveolar structural injury (pulmonary bullae and septal thickening). The severe injury observed in the LOV group was attributed to the dual effects of elevated airway and vascular pressures induced by applying harmful ventilation techniques (34, 35). The application of large tidal volumes resulted in the repetitive stretching of the lung, which led to the rupture of endothelial and epithelial cells, the filling of the alveolar space with interstitial edema, and severe destruction of the gas exchange surface (36). Furthermore, researchers have proposed that the aggregation of neutrophils in the lung forms clusters and blocks capillaries and arterioles, leading to microcirculatory obstruction in the lungs, which emphasizes the impact of microcirculatory dysfunction on tissue hypoxia caused by ARDS (37). In our present study, the LOV group exhibited the most severe leakage of neutrophils and red blood cells from damaged pulmonary microvessels into the alveolar septa and alveolar spaces, compared to the OM and LOM groups. This leakage indicated more severe microcirculatory dysfunction in the LOV group (38) (Figures 2, 3). Microcirculatory dysfunction directly affected oxygen delivery and carbon dioxide clearance, leading to more severe tissue hypoxia and metabolic disturbances, consistent with the findings of Park et al. (37).

The disruption of the pulmonary capillary barrier (lung tissue W/D ratio, BALF protein content) and the physiological dysfunction (Cdyn, BE and PaO_2_/FiO_2_) are important indicators for evaluating ARDS models (39). In our study, the W/D ratio (7.40 ± 0.56) and BALF protein content (16.01 ± 1.68 mg/mL) of the LOV group were observed to be higher than those of the OM group (6.95 ± 1.10, 14.09 ± 4.00 mg/mL) and LOM group (6.68 ± 0.56, 9.72 ± 5.19 mg/mL), indicating a significant increase in lung tissue edema and disruption of the pulmonary capillary barrier, which led to massive leakage of plasma proteins into the alveolar space. Previous studies have demonstrated a strong correlation between Cdyn values and lung tissue edema and airway resistance, which reflect alveolar tension and elasticity (40). At T6, the Cdyn of the LOV group (1.71 ± 0.25) was found to be lower than that of the OM group (2.06 ± 0.29) and the LOM group (2.02 ± 0.31), which showed a reduction of alveolar tension and elasticity in the LOV group. The main reason for these results was the enlargement of the collapse area of the lung in the LOV group, which was not stabilized by PEEP at the end of expiration, resulting in “baby lungs” (41). The application of large tidal volumes resulted in overinflation of the lung tissue, which in turn caused a reduction in Cdyn. Furthermore, it has also been demonstrated that VILI can result in excessive stretching of the alveolar walls, leading to severe disruption of the pulmonary capillary barrier and reduction of surfactant on the alveolar surface (42, 43). The low levels of surfactant on the alveolar surface resulted in a decrease in Cdyn in the LOV group. BE is an essential indicator for assessing acid–base balance in the body (44). In our experiment, the BE decrease was most pronounced in the LOV group, which suggested that VILI has a more significant impact on acidosis. This may be due to VILI exacerbating oxygenation deficits and CO_2_ retention, and increasing tidal volume in the context of lung injury did not reduce end-tidal CO_2_ levels but aggravated respiratory acidosis (45). PaO_2_/FiO_2_ is the sole quantitative indicator employed to demonstrate impaired oxygenation and the severity of ARDS (30). In our study, 4 rabbits in the LOV group (66.7%) exhibited a PaO_2_/FiO_2_ ≤ 200 mmHg, which met the criteria for moderate ARDS. In contrast, only two rabbits in the OM group (33.3%) and 1 rabbit in the LOM group (16.7%) met the criteria for moderate ARDS. Furthermore, we found that in the LOV group, the PaO_2_/FiO_2_ ratio continued to decline and remained stable after T3. In contrast, the PaO_2_/FiO_2_ ratio of animals (one rabbit in the OM group and two rabbits in the LOM group) exhibited a trend of increase after T3, but could not remain stable after the initial decline. This phenomenon indicated that the stability of the LOV group after T3 was better than that of the OM and LOM groups. Fluctuations in PaO_2_/FiO_2_ values have been observed in other studies in both the OM and LOM groups, which is a limitation of most oleic acid models. However, the specific mechanism is not yet precise (46). Therefore, it can be concluded that VILI induced in the LOV group plays a significant role in maintaining low PaO_2_/FiO_2_ values in animals with impaired lung ventilation.

Ultrasound is a non-invasive and intuitive monitoring method in ARDS research, which provides a comprehensive understanding of the evolving status of lung tissue during the development of ARDS. This provides a new perspective for the early diagnosis and treatment of ARDS (47). In our present study, the lung ultrasound score of the LOV group was significantly higher than that of the NC group at T3 and T6 (*p* < 0.01). The ultrasound scores of the OM and LOM groups did not increase significantly at T3, but increased at T6. Furthermore, the alveolar ventilation function of the LOV group was more severely impaired than that of the OM and LOM groups, as indicated by the significantly higher score of 2.33 ± 0.38 at T6 compared to 1.29 ± 0.51 in the OM group and 1.61 ± 0.26 in the LOM group. This resulted in widespread loss of ventilation. Moreover, the ultrasound results in the LOV group showed high echogenic spots and coalescent B-lines, while the OM and LOM groups mainly exhibited multiple-spaced B-lines. These findings indicated that the animals in the LOV group had experienced a severe accumulation of protein fragments and fluid within the alveoli of the lungs, accompanied by pathological changes indicative of lung consolidation (48). The results were highly consistent with the severe pulmonary edema and decreased PaO_2_/FiO_2_ observed in the LOV group in our experiment. Furthermore, we also observed a strong correlation between the ultrasound score and lung tissue wet/dry ratio, pathological score, PaO_2_/FiO_2_ ratio, and BALF protein content, which was consistent with the results of others (49–51).

It is noteworthy that, in our present study, a comparison of the results of the OM and LOM groups showed no significant synergistic effect in the LOM group, which differed from the findings of Hagawane et al., who observed a significant synergistic effect between LPS and OA (16). Our results indicated no statistical difference between the OM and LOM groups regarding lung pathological score, W/D ratio, BALF protein content, Cdyn, PaO_2_/FiO_2_ ratio and lung ultrasound score. Furthermore, the values demonstrated that the OM group with a single hit of oleic acid had more severe lung injury in the rabbit model. This discrepancy may be related to species specificity, drug dosage and usage (9, 52–54). The absence of pulmonary intravascular macrophages (PIM) in the lungs of New Zealand rabbits resulted in the low dose of LPS being insufficient to cause severe sepsis and lung injury symptoms (52–54). In addition, LPS must be administered directly to the target organ via intranasal or endotracheal routes, and intravenous administration (in our study) cannot cause tissue-specific or similar levels of lung injury, which is consistent with the views of Chen et al. (9).

Our study has several limitations. First, the 6-h modeling period only replicates the acute onset of ARDS, and the long-term effects were not assessed. Second, this study did not analyze biomarkers for lung tissue, which has significant potential for understanding the relationship between lung injury and inflammation in VILI. Future studies should implement extended intervention protocols to observe the chronic effects and investigate some biomarkers (e.g., sRAGE, IL-6, TNF-*α*) to improve our understanding of the pathophysiological mechanisms.

## 5 Conclusion

This study established a novel moderate ARDS model in New Zealand White rabbits by integrating LPS, OA, and VILI. This model successfully replicated the key features of moderate ARDS, including severe lung injury, marked pulmonary edema, and persistent hypoxemia. This model also demonstrated a high success rate and stable dynamic injury progression, accurately replicating the heterogeneity of human ARDS. Future studies should aim to validate this model with larger sample sizes and longer observation periods, and explore its potential for evaluating new ARDS treatment strategies.

## Data Availability

The original contributions presented in the study are included in the article/supplementary material, further inquiries can be directed to the corresponding authors.

## Glossary

LPS: Lipopolysaccharide
OA: Oleic acid
VILI: Ventilator-induced lung injury
ARDS: Acute respiratory distress syndrome
W/D: Lung wet-to-dry weight ratio
ICU: Intensive care unit
NHLBI: The National Heart, Lung, and Blood Institute
PEEP: Positive end-expiratory pressure
FIO_2_: Inspired oxygen fraction
RR: Respiratory rate
VT: Tidal volume
PaCO_2_: Partial pressure of carbon dioxide
CMV: Control mechanical ventilation
BALF: Bronchoalveolar lavage fluid
Cdyn: Lung compliance
HR: Heart rate
MAP: Mean arterial pressure
SPO_2_: Blood oxygen saturation
CBC: Complete blood count
HE: Hematoxylin–Eosin
WBC: White blood cell
