# Supplementary material for: Associated effects of lipopolysaccharide, oleic acid, and lung injury ventilator-induced in developing a model of moderate acute respiratory distress syndrome in New Zealand white rabbits
Source: Front Vet Sci. 2025 Mar 19;12:1477554. doi: 10.3389/fvets.2025.1477554 (PMC11963770; doi:10.3389/fvets.2025.1477554)
Supplement: Supplementary file 1 [file Data_Sheet_1.docx]

Table S1 The basic information of rabbit

| Group | Sex | Weight (kg) | Treatment | Vitality |
| --- | --- | --- | --- | --- |
| NC1 | Female | 2.3 | Saline:4 mL | Yes |
| NC2 | Male | 2.5 | Saline:4 mL | Yes |
| NC3 | Female | 2.6 | Saline:4 mL | Yes |
| NC4 | Male | 2.4 | Saline:4 mL | Yes |
| OM1 | Male | 2.4 | Saline:2 mL, OA:0.144 mL | No |
| OM2 | Male | 2.6 | Saline:2 mL, OA:0.156 mL | Yes |
| OM3 | Female | 2.5 | Saline:2 mL, OA:0.150 mL | Yes |
| OM4 | Male | 2.4 | Saline:2 mL, OA:0.144 mL | Yes |
| OM5 | Female | 2.3 | Saline:2 mL, OA:0.138 mL | Yes |
| OM6 | Female | 2.6 | Saline:2 mL, OA:0.156 mL | Yes |
| LOM1 | Female | 2.5 | LPS:1.875 mg, OA:0.150 mL | Yes |
| LOM2 | Male | 2.3 | LPS:1.725 mg, OA:0.150 mL | Yes |
| LOM3 | Male | 2.4 | LPS:1.800 mg, OA:0.144 mL | Yes |
| LOM4 | Female | 2.5 | LPS:1.875 mg, OA:0.150 mL | No |
| LOM5 | Male | 2.4 | LPS:1.800 mg, OA:0.144 mL | Yes |
| LOM6 | Female | 2.4 | LPS:1.800 mg, OA:0.144 mL | Yes |
| LOV1 | Male | 2.5 | LPS:1.875 mg, OA:0.150 mL | Yes |
| LOV2 | Male | 2.4 | LPS:1.800 mg, OA:0.144 mL | Yes |
| LOV3 | Female | 2.4 | LPS:1.800 mg, OA:0.144 mL | No |
| LOV4 | Female | 2.4 | LPS:1.800 mg, OA:0.144 mL | Yes |
| LOV5 | Female | 2.5 | LPS:1.875 mg, OA:0.150 mL | Yes |
| LOV6 | Male | 2.4 | LPS:1.800 mg, OA:0.144 mL | No |

Table S2 Results of HR (bpm)

| Time  Group | T0 | T1.5 | T3 | T4.5 | T6 |
| --- | --- | --- | --- | --- | --- |
| NC1 | 254 | 265 | 255 | 274 | 280 |
| NC2 | 254 | 260 | 245 | 269 | 258 |
| NC3 | 270 | 291 | 269 | 280 | 273 |
| NC4 | 277 | 306 | 260 | 247 | 260 |
| OM1 |  |  |  |  |  |
| OM2 | 247 | 262 | 264 | 209 | 226 |
| OM3 | 265 | 282 | 277 | 223 | 205 |
| OM4 | 220 | 203 | 228 | 225 | 220 |
| OM5 | 235 | 249 | 232 | 251 | 160 |
| OM6 | 223 | 188 | 171 | 223 | 227 |
| LOM1 | 201 | 297 | 282 | 196 | 197 |
| LOM2 | 267 | 267 | 251 | 269 | 258 |
| LOM3 | 253 | 251 | 215 | 232 | 244 |
| LOM4 |  |  |  |  |  |
| LOM5 | 247 | 210 | 221 | 223 | 221 |
| LOM6 | 267 | 254 | 237 | 259 | 304 |
| LOV1 | 203 | 220 | 206 | 192 | 241 |
| LOV2 | 289 | 232 | 223 | 232 | 238 |
| LOV3 |  |  |  |  |  |
| LOV4 | 269 | 200 | 201 | 225 | 220 |
| LOV5 | 259 | 253 | 235 | 220 | 192 |
| LOV6 |  |  |  |  |  |

Table S3 Results of MAP (mmHg)

| Time  Group | T0 | T1.5 | T3 | T4.5 | T6 |
| --- | --- | --- | --- | --- | --- |
| NC1 | 80 | 99 | 98 | 94 | 95 |
| NC2 | 91 | 94 | 91 | 92 | 97 |
| NC3 | 95 | 94 | 96 | 97 | 92 |
| NC4 | 108 | 87 | 88 | 78 | 85 |
| OM1 |  |  |  |  |  |
| OM2 | 104 | 98 | 101 | 98 | 57 |
| OM3 | 102 | 97 | 94 | 101 | 85 |
| OM4 | 84 | 87 | 82 | 83 | 84 |
| OM5 | 71 | 94 | 65 | 75 | 86 |
| OM6 | 87 | 96 | 75 | 67 | 77 |
| LOM1 | 97 | 89 | 103 | 83 | 83 |
| LOM2 | 93 | 90 | 93 | 94 | 85 |
| LOM3 | 95 | 87 | 94 | 87 | 88 |
| LOM4 |  |  |  |  |  |
| LOM5 | 93 | 74 | 86 | 70 | 65 |
| LOM6 | 92 | 79 | 74 | 71 | 63 |
| LOV1 | 100 | 89 | 92 | 86 | 55 |
| LOV2 | 99 | 92 | 92 | 94 | 78 |
| LOV3 |  |  |  |  |  |
| LOV4 | 109 | 116 | 97 | 78 | 70 |
| LOV5 | 96 | 94 | 103 | 84 | 75 |
| LOV6 |  |  |  |  |  |

Table S4 Results of WBC count (10^9/L)

| Time  Group | T0 | T1.5 | T3 | T4.5 | T6 |
| --- | --- | --- | --- | --- | --- |
| NC1 | 12.4 | 2.3 | 2.1 | 4.5 | 5.3 |
| NC2 | 11.2 | 2.8 | 2.2 | 3.6 | 5.4 |
| NC3 | 12.0 | 4.3 | 4.3 | 4.8 | 4.9 |
| NC4 | 7.1 | 2.5 | 1.2 | 2.3 | 5.2 |
| OM1 |  |  |  |  |  |
| OM2 | 9.0 | 2.2 | 1.2 | 1.0 | 2.0 |
| OM3 | 6.8 | 2.5 | 1.0 | 0.9 | 1.7 |
| OM4 | 10.5 | 2.3 | 0.6 | 0.9 | 1.8 |
| OM5 | 10.1 | 2.9 | 1.3 | 0.7 | 0.7 |
| OM6 | 12.2 | 2.7 | 0.8 | 1.4 | 3.5 |
| LOM1 | 9.6 | 2.2 | 1.2 | 0.9 | 1.9 |
| LOM2 | 7.5 | 2.8 | 1.2 | 0.9 | 1.7 |
| LOM3 | 11.7 | 4.7 | 0.6 | 0.5 | 0.9 |
| LOM4 |  |  |  |  |  |
| LOM5 | 7.4 | 2.0 | 0.6 | 0.6 | 1.0 |
| LOM6 | 7.6 | 1.2 | 0.7 | 0.5 | 0.9 |
| LOV1 | 10.2 | 1.6 | 0.5 | 0.7 | 1.1 |
| LOV2 | 11.4 | 2.0 | 1.6 | 1.4 | 3.3 |
| LOV3 |  |  |  |  |  |
| LOV4 | 8.6 | 1.9 | 0.6 | 0.8 | 1.0 |
| LOV5 | 10.8 | 2.8 | 0.6 | 1.1 | 1.9 |
| LOV6 |  |  |  |  |  |

Table S5 Lung injury score test table

| animals | Cross section | region | Visual field | Parameter | | | | |
| --- | --- | --- | --- | --- | --- | --- | --- | --- |
|  |  |  |  | A | B | C | D | E |
|  |  | Top left | 1 |  |  |  |  |  |
|  |  |  | 2 |  |  |  |  |  |
|  |  |  | 3 |  |  |  |  |  |
|  |  |  | 4 |  |  |  |  |  |
|  |  |  | 5 |  |  |  |  |  |
|  |  | Top right | 1 |  |  |  |  |  |
|  |  |  | 2 |  |  |  |  |  |
|  |  |  | 3 |  |  |  |  |  |
|  |  |  | 4 |  |  |  |  |  |
|  |  |  | 5 |  |  |  |  |  |
|  |  | Bottom left | 1 |  |  |  |  |  |
|  |  |  | 2 |  |  |  |  |  |
|  |  |  | 3 |  |  |  |  |  |
|  |  |  | 4 |  |  |  |  |  |
|  |  |  | 5 |  |  |  |  |  |
|  |  | Bottom right | 1 |  |  |  |  |  |
|  |  |  | 2 |  |  |  |  |  |
|  |  |  | 3 |  |  |  |  |  |
|  |  |  | 4 |  |  |  |  |  |
|  |  |  | 5 |  |  |  |  |  |

Note: A total of 22 rabbits were used, and from each rabbit, the lower lobe of the left lung was taken for HE staining, and five sections were intercepted according to the direction of gravity. Each slice was divided into four regions: upper left, upper right, lower left and lower right. Each region randomly selected five horizons to pathological scroing (400 X)

Table S6 Summary of pathological score

| Group | Parameter | | | | | Pathological score |
| --- | --- | --- | --- | --- | --- | --- |
|  | A | B | C | D | E |  |
| NC1 | 0.10 | 0.01 | 0.00 | 0.33 | 0.80 | 0.0605 |
| NC2 | 0.33 | 0.24 | 0.01 | 0.20 | 0.66 | 0.1275 |
| NC3 | 0.22 | 0.16 | 0.00 | 0.26 | 0.50 | 0.0946 |
| NC4 | 0.44 | 0.24 | 0.01 | 0.05 | 0.61 | 0.1380 |
| OM1 |  |  |  |  |  |  |
| OM2 | 1.59 | 0.92 | 0.38 | 1.60 | 0.93 | 0.6040 |
| OM3 | 1.54 | 1.15 | 0.17 | 1.11 | 1.63 | 0.5912 |
| OM4 | 1.47 | 1.31 | 0.2 | 1.11 | 1.54 | 0.5999 |
| OM5 | 1.51 | 0.90 | 0.08 | 1.14 | 1.27 | 0.5388 |
| OM6 | 1.39 | 1.05 | 0.14 | 1.29 | 1.40 | 0.5531 |
| LOM1 | 1.28 | 0.91 | 0.08 | 1.60 | 0.90 | 0.5190 |
| LOM2 | 1.15 | 1.17 | 0.07 | 1.51 | 0.99 | 0.5242 |
| LOM3 | 1.40 | 1.06 | 0.14 | 1.75 | 0.90 | 0.5787 |
| LOM4 |  |  |  |  |  |  |
| LOM5 | 1.41 | 0.93 | 0.13 | 1.79 | 0.78 | 0.5622 |
| LOM6 | 1.14 | 1.15 | 0.28 | 1.85 | 0.99 | 0.5579 |
| LOV1 | 1.94 | 1.68 | 0.89 | 1.83 | 1.82 | 0.8500 |
| LOV2 | 1.79 | 1.62 | 0.42 | 1.94 | 1.54 | 0.7808 |
| LOV3 |  |  |  |  |  |  |
| LOV4 | 1.95 | 1.49 | 0.53 | 1.87 | 1.60 | 0.7983 |
| LOV5 | 1.76 | 1.62 | 1.00 | 1.85 | 1.66 | 0.8115 |
| LOV6 |  |  |  |  |  |  |

Table S7 Lung tissue wet-to-dry (W/D) ratio and BALF protein content

| Group | BALF protein content(mg/mL) | Lung Tissue W/D Ratio |
| --- | --- | --- |
| NC1 | 0.06 | 4.97 |
| NC2 | 0.86 | 5.19 |
| NC3 | 2.28 | 5.33 |
| NC4 | 1.05 | 5.29 |
| OM1 |  |  |
| OM2 | 17.44 | 8.15 |
| OM3 | 11.21 | 5.63 |
| OM4 | 9.31 | 6.00 |
| OM5 | 18.72 | 7.19 |
| OM6 | 13.76 | 7.76 |
| LOM1 | 14.31 | 7.35 |
| LOM2 | 4.36 | 6.56 |
| LOM3 | 10.48 | 6.41 |
| LOM4 |  |  |
| LOM5 | 15.08 | 7.12 |
| LOM6 | 4.35 | 5.95 |
| LOV1 | 16.78 | 6.66 |
| LOV2 | 16.95 | 7.86 |
| LOV3 |  |  |
| LOV4 | 13.50 | 7.8 |
| LOV5 | 16.82 | 7.27 |
| LOV6 |  |  |

Table S8 Results of Cdyn (mL/cmH2O)

| Time  Group | T0 | T3 | T6 |
| --- | --- | --- | --- |
| NC1 | 3.00 | 2.80 | 2.60 |
| NC2 | 2.83 | 2.80 | 2.70 |
| NC3 | 2.20 | 2.70 | 2.50 |
| NC4 | 2.50 | 3.10 | 3.30 |
| OM1 |  |  |  |
| OM2 | 3.00 | 2.70 | 2.00 |
| OM3 | 2.90 | 2.70 | 2.00 |
| OM4 | 2.50 | 2.08 | 2.10 |
| OM5 | 2.50 | 2.50 | 1.80 |
| OM6 | 3.57 | 2.50 | 2.40 |
| LOM1 | 2.60 | 2.10 | 1.80 |
| LOM2 | 3.50 | 2.50 | 2.50 |
| LOM3 | 3.20 | 2.40 | 2.00 |
| LOM4 |  |  |  |
| LOM5 | 2.80 | 3.10 | 1.70 |
| LOM6 | 2.40 | 1.92 | 2.08 |
| LOV1 | 3.20 | 2.40 | 1.60 |
| LOV2 | 3.57 | 2.10 | 1.67 |
| LOV3 |  |  |  |
| LOV4 | 3.57 | 2.78 | 2.08 |
| LOV5 | 2.50 | 2.08 | 1.50 |
| LOV6 |  |  |  |

Table S9 Results of PaO2/FiO2 (mmHg)

| Time  Group | T0 | T3 | T6 |
| --- | --- | --- | --- |
| NC1 | 430 | 415 | 457 |
| NC2 | 470 | 467 | 425 |
| NC3 | 422 | 414 | 398 |
| NC4 | 343 | 360 | 370 |
| OM1 |  |  |  |
| OM2 | 472 | 453 | 137 |
| OM3 | 370 | 395 | 437 |
| OM4 | 477 | 422 | 285 |
| OM5 | 358 | 263 | 103 |
| OM6 | 365 | 356 | 263 |
| LOM1 | 283 | 410 | 132 |
| LOM2 | 445 | 280 | 235 |
| LOM3 | 448 | 423 | 433 |
| LOM4 |  |  |  |
| LOM5 | 428 | 262 | 210 |
| LOM6 | 418 | 370 | 385 |
| LOV1 | 408 | 256 | 160 |
| LOV2 | 453 | 440 | 132 |
| LOV3 |  |  |  |
| LOV4 | 490 | 357 | 190 |
| LOV5 | 398 | 363 | 168 |
| LOV6 |  |  |  |

Table S10 Results of BE (mEq/L)

| Time  Group | T0 | T3 | T6 |
| --- | --- | --- | --- |
| NC1 | -3.00 | -2.00 | -3.00 |
| NC2 | -2.00 | 1.00 | -1.00 |
| NC3 | 3.00 | 2.00 | 2.00 |
| NC4 | -2.00 | -5.00 | -4.00 |
| OM1 |  |  |  |
| OM2 | -2.00 | -4.00 | -20.00 |
| OM3 | -2.00 | -2.00 | -2.00 |
| OM4 | -1.00 | .00 | 3.00 |
| OM5 | -3.00 | -5.00 | -2.00 |
| OM6 | -3.00 | -7.00 | -10.00 |
| LOM1 | 3.00 | 2.00 | -13.00 |
| LOM2 | -1.00 | -8.00 | -9.00 |
| LOM3 | -3.00 | -4.00 | -2.00 |
| LOM4 |  |  |  |
| LOM5 | -3.00 | -7.00 | -11.00 |
| LOM6 | .00 | -8.00 | -12.00 |
| LOV1 | 1.00 | -5.00 | -23.00 |
| LOV2 | -2.00 | -10.00 | -14.00 |
| LOV3 |  |  |  |
| LOV4 | -2.00 | -9.00 | -15.00 |
| LOV5 | -2.00 | -7.00 | -14.00 |
| LOV6 |  |  |  |
